# Role of *Lactobacillus* Species in the Intermediate Vaginal Flora in Early Pregnancy: A Retrospective Cohort Study

**DOI:** 10.1371/journal.pone.0144181

**Published:** 2015-12-11

**Authors:** Alex Farr, Herbert Kiss, Michael Hagmann, Susanne Machal, Iris Holzer, Verena Kueronya, Peter Wolf Husslein, Ljubomir Petricevic

**Affiliations:** 1 Department of Obstetrics and Gynaecology, Division of Obstetrics and Fetomaternal Medicine, Medical University of Vienna, Vienna, Austria; 2 Section for Medical Statistics, Centre of Medical Statistics, Informatics and Intelligent Systems, Medical University of Vienna, Vienna, Austria; University of Missouri, UNITED STATES

## Abstract

**Background:**

Poor obstetrical outcomes are associated with imbalances in the vaginal flora. The present study evaluated the role of vaginal *Lactobacillus* species in women with intermediate vaginal flora with regard to obstetrical outcomes.

**Methods:**

We retrospectively analysed data from all women with singleton pregnancies who had undergone routine screening for asymptomatic vaginal infections at our tertiary referral centre between 2005 and 2014. Vaginal smears were Gram-stained and classified according to the Nugent scoring system as normal flora (score 0–3), intermediate vaginal flora (4–6), or bacterial vaginosis (7–10). Only women with intermediate vaginal flora were investigated. Women with a Nugent score of 4 were categorised into those with and without Lactobacilli. Follow-up smears were obtained 4–6 weeks after the initial smears. Descriptive data analysis, the Welch’s *t*-test, the Fisher’s exact test, and multiple regression analysis with adjustment for confounders were performed. Gestational age at delivery and birth weight were the outcome measures.

**Results:**

At antenatal screening, 529/8421 women presented with intermediate vaginal flora. Amongst these, 349/529 (66%) had a Nugent score of 4, 94/529 (17.8%) a Nugent score of 5, and 86/529 (16.2%) a Nugent score of 6. Amongst those with a Nugent score of 4, 232/349 (66.5%) women were in the Lactobacilli group and 117/349 (33.5%) in the Non-Lactobacilli group. The preterm delivery rate was significantly lower in the Lactobacilli than in the Non-Lactobacilli group (OR 0.34, CI 0.21–0.55; *p*<0.001). Mean birth weight was 2979 ± 842 g and 2388 ± 1155 g in the study groups, respectively (MD 564.12, CI 346.23–781.92; *p*<0.001). On follow-up smears, bacterial vaginosis rates were 9% in the Lactobacilli and 7.8% in the Non-Lactobacilli group.

**Conclusions:**

The absence of vaginal *Lactobacillus* species and any bacterial colonisation increases the risks of preterm delivery and low birth weight in women with intermediate vaginal flora in early pregnancy.

## Introduction

Despite recent achievements in perinatal care, the prevention of preterm delivery (PTD) remains a major challenge in modern obstetrics [1]. Healthy vaginal flora, dominated by *Lactobacillus* species (spp.), plays an important role in the protection against genital infections, which are considered a frequent cause of PTD [2]. Imbalances of the vaginal flora caused by a reduction in the proportion of *Lactobacillus* spp. can lead to an overgrowth of mixed anaerobic bacteria, which can ultimately result in bacterial vaginosis (BV) [3]. Since the effect of treating pregnant women with an abnormal vaginal flora was introduced, BV has gained interest and is now a well-known risk factor for ascending infections and subsequent PTD [4]. However, the overall risk reduction of PTD through antibiotic treatment of an abnormal flora was disproved by large and well-designed studies in a general obstetrical population [5,6].

At present, little is known about the role of the intermediate vaginal flora, which can either be regarded as the transition from a healthy to an abnormal flora or as the transition from abnormal flora back to a healthy vaginal microbiota [7]. The intermediate vaginal flora constitutes a highly heterogeneous group that may or may not include vaginal *Lactobacillus* spp. and that may or may not be characterised by the presence of anaerobic bacteria [8,9]. In 2007, a meta-analysis reported that the presence of intermediate vaginal flora was not associated with PTD, late miscarriage, maternal or neonatal infection, or perinatal mortality [10]. In contrast, an observational study by Hay et al. [11] reported a high rate of late miscarriage (16–24 gestational weeks) in women with intermediate vaginal flora at an examination before 16 gestational weeks. Additionally, another study reported an association of PTD with partial BV, which is defined as streaks of BV flora in a smear that has areas of normal flora [12].

Considering that the association between intermediate vaginal flora and PTD is unclear, the present study aimed to evaluate the role of vaginal *Lactobacillus* spp. in women with intermediate vaginal flora, according to the Nugent score in early pregnancy.

## Materials and Methods

### Ethics statement

The study was performed with the approval of the ethics committee of the Medical University of Vienna (Amendment to Protocol Number 1101/2014) in accordance with the Declaration of Helsinki and the guidelines of Good Clinical Practice, supported by the Head of the Institute. Due to the retrospective character of the study, written inform consent was not obtained. Patient records and data were anonymised and de-identified prior to analysis.

### Setting and procedure

In this study, data of all women who presented with singleton pregnancies at the Medical University of Vienna, Department of Obstetrics and Gynaecology, between 1 January 2005 and 31 December 2014 were retrospectively analysed. Our centre is the largest tertiary referral centre in Austria, and it specialises in high-risk pregnancy care. The centre performs approximately 3000 deliveries per year and receives referrals from the entire Central-Eastern Europe.

Pregnancy care included a prenatal consultation, where women registered for a planned delivery at our department between 10 + 0 (10 weeks plus 0 days) and 16 + 0 (16 weeks plus 0 days) gestational weeks. Women underwent routine screening for asymptomatic vaginal infections. Additionally, foetal nuchal translucency was measured, and medical and obstetric history was taken [13]. All women except those with a normal flora (Nugent score 0–3) were asked to undergo a follow-up smear between 14 + 0 (14 weeks plus 0 days) and 22 + 0 (22 weeks plus 0 days) gestational weeks at our department. Further consultations were compulsory and were performed at predetermined time points at obstetric offices, following the official Austrian welfare programme that ensures the health precautions for pregnant women and their foetuses [14].

Vaginal smears were obtained after vaginal fluid collection with sterile swabs from the lateral vaginal wall and posterior fornix vaginae. All smears were Gram-stained and microscopically analysed by one of four biomedical laboratory assistants, specialised in gynaecological cytopathology, at a laboratory that is certified according to DIN EN ISO 9001:2008. (Regular quality management trainings are compulsory for all laboratory assistants, who together analyse more than 5300 Gram-stained smears per year). The protocol involved classification of the vaginal flora as described by Nugent et al. [9]. Nugent scores of 0–3 were considered to indicate normal vaginal flora, 4–6 to indicate intermediate vaginal flora, and 7–10 to indicate BV. According to the Nugent scoring system, a Nugent score of 4 included vaginal flora with and that without *Lactobacillus* spp. The presence or absence of *Candida albicans* and *Trichomonas vaginalis* were assessed by a microbial identification test using DNA probe technology (BD Affirm^™^ VP III; Becton Dickinson Co., Sparks, MD, USA).

Following our clinical protocol, women with normal or intermediate vaginal flora did not receive treatment. The treatment of BV included clindamycin 2% vaginal cream for 6 days in cases of a primary infection, oral clindamycin (0.3 g) twice daily for 7 days in cases of recurrent BV infection, local clotrimazole (0.1 g) for 6 days in cases of vaginal candidiasis, and local metronidazole (0.5 g) for 7 days in cases of trichomoniasis [15]. Antibiotic treatment was followed by vaginally applied *Lactobacillus* spp. for 6 days to rebuild the physiological flora [16].

### Study groups

The study population included women who had registered for a planned delivery, underwent routine infection screening, and had intermediate vaginal flora. Screened women included those with chronic diseases, as well as those with comorbidities and poor obstetric histories. However, only women without signs of vaginal infection (no conspicuous redness, no discharge or vaginal itch) were eligible for the study. Indeed, women with normal vaginal flora (Nugent score 0–3), as well as those with BV (Nugent score 7–10), trichomoniasis, and/or candidiasis on initial screening smears were not eligible for inclusion into the study. Furthermore, women who were antenatally referred from other hospitals owing to imminent PTD and those who did not undergo the antenatal screening programme were not included in the study population.

In women with a Nugent score of 4, we discriminated between those with and those without vaginal *Lactobacillus* spp., viz. the Lactobacilli group and Non-Lactobacilli group. The Lactobacilli group was defined as those with intermediate vaginal flora (Nugent score 4) with colonisation by bacteria including vaginal *Lactobacillus* spp. The Non-Lactobacilli group was defined as those with intermediate vaginal flora (Nugent score 4) without any bacterial colonisation or *Lactobacillus* spp., showing only epithelial cells on the examined smears. We did not further evaluate the role of *Lactobacillus* spp. in women with Nugent scores of 5 and 6, since the overgrowth of mixed anaerobic bacteria may have influenced our results in these groups [9]. We also considered that effects that are related to BV may be weaker in women with a Nugent score of 4 than in those with Nugent scores of 5 and 6 [17].

### Outcome measures

The gestational age at delivery was considered the primary outcome variable, and was recorded as term delivery at or after 37 + 0 (37 weeks plus 0 days) gestational weeks. PTD was defined as spontaneous delivery at or before 36 + 6 (36 weeks plus 6 days) gestational weeks due to preterm premature rupture of the membranes and/or preterm labour. The secondary outcome variables were PTD and low birth weight defined as a weight of less than 2500 g. Stillbirth was defined as the term or PTD of an infant that had died in utero (Apgar scores of 0/0/0). Data were extracted from obstetric databases, patient charts, and microbiologic reports.

### Statistical analysis

Demographic information was summarised and displayed using descriptive statistics. Continuous data are presented as mean ± standard deviation (SD), unless stated otherwise. Discrete data are presented as number (percentage). The Welch’s *t*-test was used to compare continuous data, and the Fisher’s exact test was used to compare categorical data. Logistic or linear regression was used to determine the adjusted effects of variables. The multiple regression model included confounding variables that were unequally distributed between the groups and were compared and considered to have an impact on the end-point. The decision to adjust for a potential confounder was based on Welch’s *t*-test for continuous variables and Fisher’s exact test for dichotomous variables. All statistical analyses were performed using R-Project for Statistical Computing, version 3.1.3 (http://www.r-project.org; R Development Core Team, Boston, MA) and SPSS Statistics, version 23.0 (IBM, Armonk, NY). Patient charts were electronically reviewed using the PIA Fetal Database, version 5.6.16.917 (GE Viewpoint, Munich, Germany). A two-sided *p*-value < 0.05 was considered to indicate statistical significance in all the tests.

## Results

A total of 8421 asymptomatic women with singleton pregnancies presented for routine antenatal screening at our tertiary referral centre between 2005 and 2014. Intermediate vaginal flora was identified on the examined screening smears of 529/8421 (6.3%) women. In this group, 349/529 (66%) women had a Nugent score of 4, 94/529 (17.8%) had a Nugent score of 5, and 86/529 (16.2%) had a Nugent score of 6. In women with a Nugent score of 4, 232/349 (66.5%) women were assigned to the Lactobacilli group. The Non-Lactobacilli group consisted of 117/349 (33.5%) women with a Nugent score of 4 due to the total absence of any bacterial colonisation, including *Lactobacillus* spp. According to our routine protocol, women in neither study group received any treatment for their vaginal microbiota. The baseline variables of the 529 study participants with intermediate vaginal flora, according to the Nugent score, are presented in Table 1.

**Table 1:** Baseline variables of the 529 women with intermediate vaginal flora, according to the Nugent score.

Variable	Intermediate vaginal flora (N = 529)			Total (N = 529)
	Nugent score 4	Nugent score 5	Nugent score 6	
	Mean ± SD	Mean ± SD	Mean ± SD	Mean ± SD)
	N (%)	N (%)	N (%)	N (%)
**Participants**	349 (66)	94 (17.8)	86 (16.2)	529 (100)
**Age at delivery (years)**	30.9 ± 6.3	30.3 ± 6.9	30.6 ± 6.4	30.7 ± 6.4
**Parity**				
Primiparae	156 (44.7)	31 (33)	30 (34.9)	217 (41)
Multiparae	193 (55.3)	63 (67)	56 (65.1)	312 (59)
**History of PTD**	1 (0.3)	0 (0)	0 (0)	1 (0.2)
**Tertiary education** ‡	17 (4.9)	4 (4.3)	5 (5.8)	26 (4.9)
**Tobacco smoking**	44 (12.6)	20 (21.3)	16 (18.6)	80 (15.1)

N = number, SD = standard deviation, PTD = preterm delivery

^‡^defined by academic degree

The cohort of women with intermediate vaginal flora at antenatal screening delivered at a mean of 36.6 ± 4.9 gestational weeks, corresponding to a PTD rate of 30.4%. Among women with a Nugent score of 4, those with a score of 5, and those with a score of 6, the PTD rates were 32.7%, 27.7%, and 24.4%, respectively. The detailed obstetrical outcomes of all the women with intermediate vaginal flora are presented in Table 2. In the group with a Nugent score of 4, women in the Lactobacilli group had a mean gestational age at delivery of 37.5 ± 4.9 weeks, compared to 34.3 ± 5.8 weeks in the Non-Lactobacilli group (mean difference [MD] 3.18, confidence interval [CI] 1.99–4.36; *p* < 0.001). Rates of PTD in these groups were 24.1% and 49.6%, respectively (odds ratio [OR] 0.33, CI 0.19–0.53; *p* < 0.001).

**Table 2:** Detailed obstetrical outcomes of the 529 women with intermediate vaginal flora according to the Nugent score.

Variable	Category	Intermediate vaginal flora (N = 529)						Total (N = 529)	
		Nugent score 4 (N = 349)		Nugent score 5 (N = 94)		Nugent score 6 (N = 86)			
		N (%)	95% CI	N (%)	95% CI	N (%)	95% CI	N (%)	95% CI
**Pregnancy outcome**	Live birth	346 (99.1)	98.57–100	94 (17.8)	100–100	84 (97.7)	95.35–100	524 (99.1)	98.49–99.88
	Stillbirth	3 (0.9)	0.29–1.85	0 (0)	0–1.7	2 (2.3)	0–4.74	5 (0.9)	0.38–1.77
**Prematurity**	Preterm delivery	114 (32.7)	27.79–37.77	26 (27.7)	19.15–36.76	21 (24.4)	16.28–33.76	161 (30.4)	26.47–34.41
	No preterm delivery	235 (67.3)	62.46–72.44	68 (72.3)	63.83–81.44	65 (75.6)	67.44–84.92	368 (69.6)	65.6–73.54
**Mode of delivery**	Vaginal delivery†	172 (49.3)	43.84–54.76	50 (53.2)	43.62–63.99	50 (58.1)	48.84–69.64	272 (51.4)	47.07–55.96
	Caesarean section	165 (47.3)	41.83–52.75	43 (45.7)	36.17–56.54	35 (40.7)	31.4–52.2	243 (46)	41.59–50.48
	Instrumental delivery	12 (3.4)	0–8.91	1 (1.1)	0–11.86	1 (1.2)	0–12.67	14 (2.6)	0–6.23
**Gestational week at delivery**	< 23w 0d	7 (2)	0–6.91	4 (4.2)	0–13.59	1 (1.2)	0–9.8	12 (2.3)	0–6.23
	23w 0d–27w 6d	33 (9.5)	4.87–14.36	9 (9.6)	2.13–18.91	2 (2.3)	0–10.96	44 (8.3)	4.73–12.28
	28w 0d–31w 6d	26 (7.4)	2.87–12.35	1 (1.1)	0–10.4	6 (7)	0–15.61	33 (6.2)	2.65–10.2
	32w 0d–36w 6d	48 (13.8)	9.17–18.65	12 (12.8)	5.32–22.1	12 (13.9)	5.81–22.59	72 (13.6)	10.02–17.57
	≥ 37w 0d	235 (67.3)	62.75–72.24	68 (72.3)	64.89–81.67	65 (75.6)	67.44–84.22	368 (69.6)	65.97–73.53
**Birth weight** *	< 500 g	5 (1.4)	0–6.14	3 (3.2)	0–11.01	1 (1.2)	0–10.53	9 (1.7)	0–5.33
	500–999 g	28 (8.1)	3.75–12.77	9 (9.6)	2.13–17.39	2 (2.4)	0–11.71	39 (7.4)	3.8–11.03
	1000–1499 g	22 (6.3)	2.02–11.04	2 (2.1)	0–9.94	3 (3.5)	0–12.88	27 (5.1)	1.52–8.75
	1500–2499 g	43 (12.4)	8.07–17.09	7 (7.4)	0–15.26	16 (18.8)	10.59–28.18	66 (12.6)	8.94–16.17
	≥ 2500 g	249 (71.8)	67.44–76.46	73 (77.7)	70.21–85.47	63 (74.1)	65.88–83.47	385 (73.2)	69.58–76.81

N = number, CI = confidence interval

^†^including vaginal breech delivery

*no birth weight available for 2 of the 349 infants (Nugent score 4), 1 of the 86 infants (Nugent score 6), and 3 of the 529 infants (Total)

**Fig 1:**
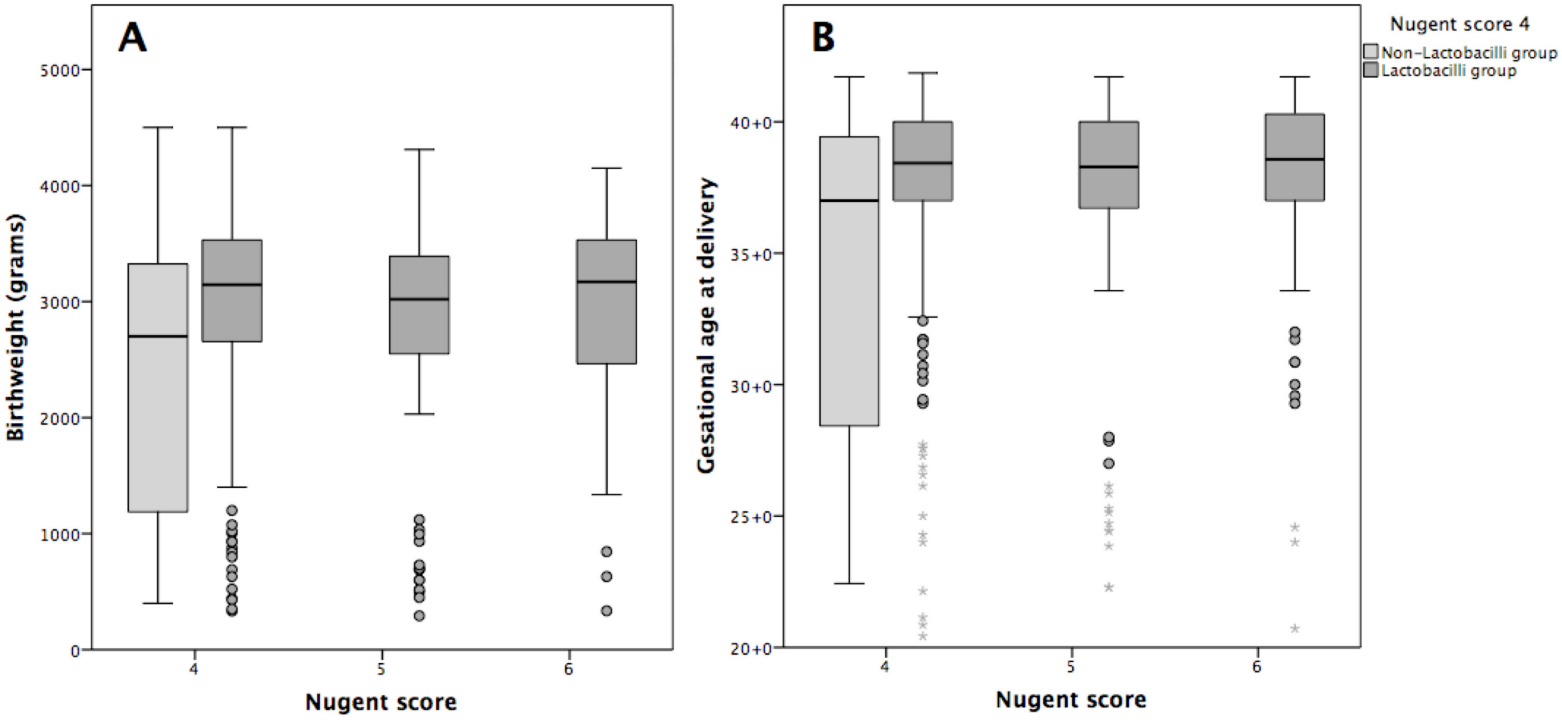
Birth weight (A) and gestational age (B) at delivery in 529 women with intermediate vaginal flora.

The detailed obstetrical outcomes of the 349 women with intermediate vaginal flora and a Nugent score of 4 are presented in Table 3. The mean birth weight at delivery in the observed population with intermediate vaginal flora was 2813 ± 971 g. Infants of the Lactobacilli group had a mean birth weight of 2979 ± 842 g, compared to 2388 ± 1155 g in those of the Non-Lactobacilli group (MD 591.66, CI 352.68–830.64; *p* < 0.001). Women in the Lactobacilli group less frequently gave birth to infants with a birth weight of less than 2500 g than did the Non-Lactobacilli group (20.7% versus 42.8%; OR 0.35, CI 0.21–0.58; *p* < 0.001; Fig 1).

**Table 3:** Detailed obstetrical outcomes of the 349 women with intermediate vaginal flora and a Nugent score of 4.

Variable	Category	Intermediate vaginal flora and Nugent score of 4 (N = 349)			
		Lactobacilli group (N = 232)		Non-Lactobacilli group (N = 117)	
		N (%)	95% CI	N (%)	95% CI
**Pregnancy outcome**	Live birth	231 (99.6)	99.14–100	115 (98.3)	96.58–100
	Stillbirth	1 (0.4)	0–1.16	2 (1.7)	0–3.49
**Prematurity**	Preterm delivery	56 (24.1)	18.97–29.82	58 (49.6)	41.03–59.37
	No preterm delivery	176 (75.9)	70.69–81.54	59 (50.4)	41.88–60.23
**Mode of delivery**	Vaginal delivery†	120 (51.7)	45.26–58.57	52 (44.4)	35.9–54.23
	Caesarean section	102 (44)	37.5–50.81	63 (53.9)	45.3–63.63
	Instrumental delivery	10 (4.3)	0–11.15	2 (1.7)	0–11.49
**Gestational week at delivery**	< 23w 0d	4 (1.7)	0–6.97	3 (2.6)	0–12.11
	23w 0d–27w 6d	10 (4.3)	0–9.55	23 (19.7)	11.11–29.2
	28w 0d–31w 6d	9 (3.9)	0–9.12	17 (14.5)	5.98–24.08
	32w 0d–36w 6d	33 (14.2)	9.05–19.47	15 (12.8)	4.27–22.37
	≥ 37w 0d	176 (75.9)	70.69–81.11	59 (50.4)	41.88–59.97
**Birth weight** *	< 500 g	4 (1.7)	0–6.78	1 (0.9)	0–9.9
	500–999 g	8 (3.5)	0–8.5	20 (17.4)	8.7–26.42
	1000–1499 g	6 (2.6)	0–7.64	16 (13.9)	5.22–22.94
	1500–2499 g	30 (12.9)	8.19–17.99	13 (11.3)	2.61–20.34
	≥ 2500 g	184 (79.3)	74.57–84.37	65 (56.5)	47.83–66.13

N = number, CI = confidence interval

^†^including vaginal breech delivery

*no birth weight available for 2 of 349 infants (Non-Lactobacilli group)

In order to determine the factors that should be included as potential confounders in the multiple regression model, including maternal age, parity, tertiary education, tobacco smoking, and history of PTD, the Fisher’s exact test and Welch’s *t*-test were performed. Parity was the only significant risk factor for PTD on comparing the Lactobacilli and Non-Lactobacilli groups (OR 2.27, CI 1.41–3.67; *p* < 0.001). Additionally, parity was a significant risk factor for PTD on comparing the overall group of women with intermediate vaginal flora and the Non-Lactobacilli group (OR 2.45, CI 1.58–3.81; *p* < 0.001). After adjusting for parity, the absence of *Lactobacillus* spp. and other bacteria was found to have a significant effect on the gestational age at delivery (MD 3.09, CI 2.03–4.16; *p* < 0.001) and birth weight (MD 564.12, CI 346.23–781.92; *p* < 0.001) in the 529 women with intermediate vaginal flora (Table 4).

**Table 4:** Univariate and multivariate analyses for the obstetrical outcomes of the 529 women with intermediate vaginal flora.

	**Lactobacilli group** * **versus Non-Lactobacilli group (Ref.)** *			**All Intermediate** ** **versus Non-Lactobacilli group (Ref.)** *		
**Univariate analysis**	Mean difference, Odds ratio	95% CI	*p*-value	Mean difference, Odds ratio	95% CI	*p*-value
**Gestational age at delivery (weeks)** †	3.18	1.99–4.36	*< 0*.*001*	2.97	1.82–4.11	*< 0*.*001*
**Delivery < 37w 0d vs. ≥ 37w 0d** ††	0.33	0.19–0.53	*< 0*.*001*	0.34	0.22–0.53	*< 0*.*001*
**Birth weight (grams)** †	591.66	352.68–830.64	*< 0*.*001*	544.69	315.31–774.07	*< 0*.*001*
**Birth weight < 2500 g vs. ≥ 2500 g** ††	0.35	0.21–0.58	*< 0*.*001*	0.38	0.24–0.59	*< 0*.*001*
**Multivariate analysis**	Mean difference, Odds ratio	95% CI	*p*-value	Mean difference, Odds ratio	95% CI	*p*-value
**Gestational age at delivery (weeks)** ‡	3.09	2.03–4.16	*< 0*.*001*	2.83	1.84–3.82	*< 0*.*001*
**Delivery < 37w 0d vs. ≥ 37w 0d** ‡‡	0.34	0.21–0.55	*< 0*.*001*	0.36	0.24–0.56	*< 0*.*001*
**Birth weight (grams)** ‡	564.12	346.23–781.92	*< 0*.*001*	506.49	308.14–704.83	*< 0*.*001*
**Birth weight < 2500 g vs. ≥ 2500 g** ‡‡	0.37	0.23–0.61	*< 0*.*001*	0.40	0.26–0.63	*< 0*.*001*

CI = confidence interval, Ref. = reference

Outcome variables:

^†^continuous data using the Welch’s t-test,

^††^categorical data using the Fisher’s exact test,

^‡^linear regression, adjusted for parity as an associated risk factor for PTD,

^‡‡^logistic regression adjusted for parity as an associated risk factor for PTD

*Nugent score 4 (N = 349)

**Nugent score 4–6 (N = 529)

**Fig 2:**
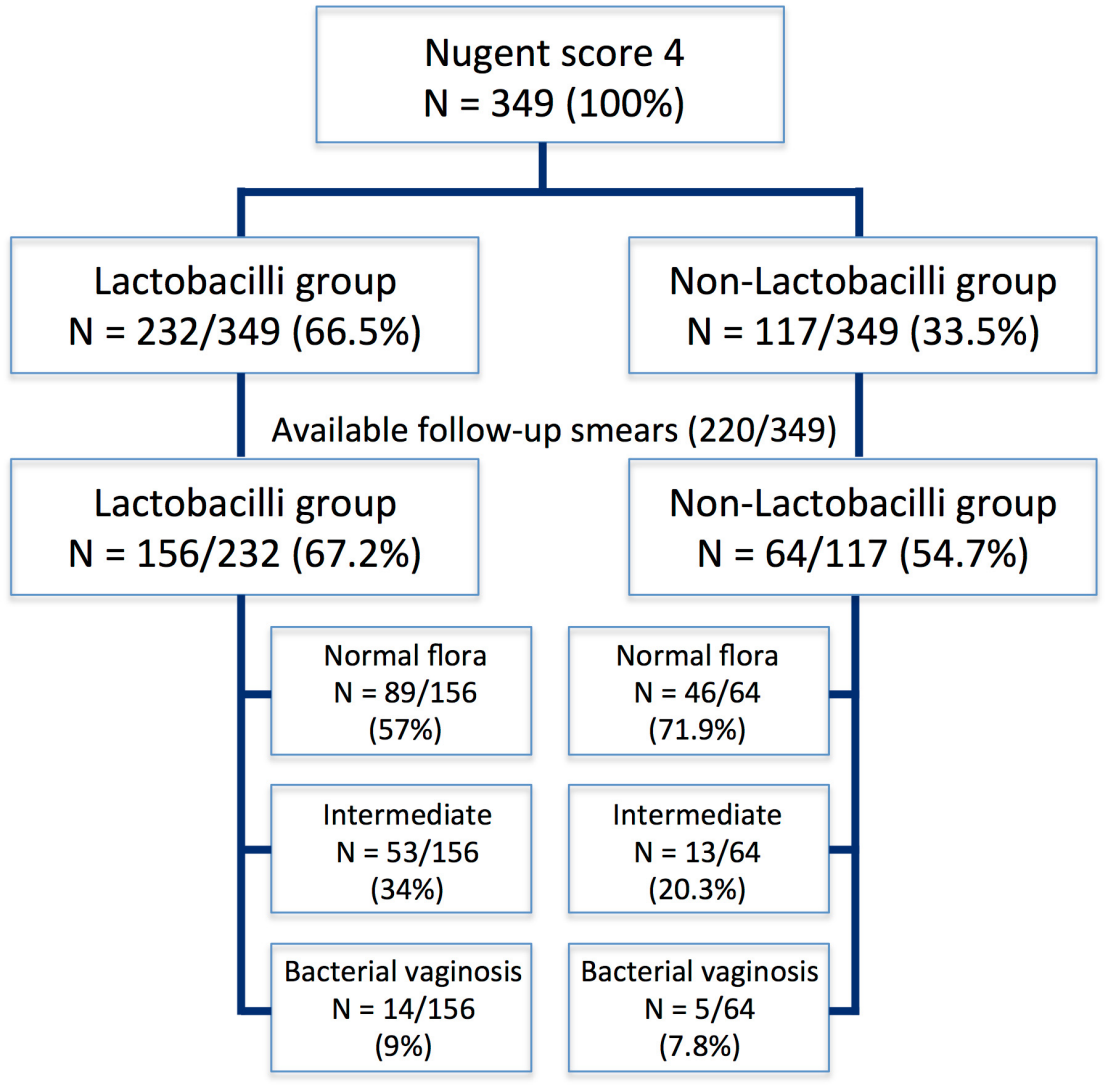
Vaginal follow-up smears of the 349 women with intermediate vaginal flora and a Nugent score of 4 at the initial screening.

Follow-up smears were available for 220/349 (63%) women who had an initial Nugent score of 4, and the rest of the women were lost to follow-up. These women included 156/232 (67.2%) in the Lactobacilli group and 64/117 (54.7%) in the Non-Lactobacilli group. As shown Fig 2, 89/156 (57%) women of the Lactobacilli group developed a normal vaginal flora, whereas 14/156 (9%) proceeded to BV. In the Non-Lactobacilli group, normal flora and BV were found in the follow-up smears of 46/64 (71.9%) and 5/64 (7.8%) women, respectively.

## Discussion

The present study aimed to evaluate the role of vaginal *Lactobacillus* spp. in women with intermediate vaginal flora, as very little information is available on this matter in the literature [8]. In debates on the effectiveness of antenatal screening programmes for abnormal vaginal flora, studies refer mostly to BV as a dangerous condition that triggers an ascending infection through an imbalance in the vaginal flora, increasing the risk for spontaneous PTD in affected women [6,13,18]. In addition, studies have reported that intermediate vaginal flora may play a role in the infection-related aetiology of PTD through not only a transition towards BV, but also a reduction in the proportion of *Lactobacillus* spp. [19,20]. In our study, among women with intermediate vaginal flora and a Nugent score of 4, we compared those with and those without vaginal *Lactobacillus* spp. and observed an association between the absence of vaginal *Lactobacillus* spp., PTD and low birth weight.

In our study, the inclusion of women with intermediate vaginal flora was based on the scoring system of Nugent et al. [9], which estimates the proportion of bacterial morphotypes under the microscope. We found a rate of 6.3% (529/8421) with intermediate vaginal flora and a Nugent score of 4–6, which is less than the incidence of 10–21% reported in other studies that evaluated vaginal flora in asymptomatic women during the first trimester of pregnancy [12,20,21]. This difference might be because the socioeconomic status of most of the women was higher in our study than in the previous study. Moreover, the women might have received preconception treatment for abnormal flora. The majority of patients with intermediate vaginal flora in our study had a Nugent score of 4 (66%).

Despite results from a large prospective study that reported PTD in women with a deficiency of *Lactobacillus* spp. at between 25 and 35 gestational weeks, data with regard to the Nugent scoring system are lacking [12]. To our knowledge, our study is the first to evaluate the Nugent scoring system in women with intermediate vaginal flora and to show lower rates of PTD in women with Nugent scores of 5 and 6 than in those with a Nugent score of 4. As shown in Fig 1 and as listed in Table 2, we found that women with a Nugent score of 6 had an increased gestational age at delivery and birth weight compared to the overall group or those with a Nugent score of 4. We had expected to find a high prevalence of PTD among women with high Nugent scores that tended towards BV, such as a Nugent score of 6. In the absence of adjustments for potential confounders, the inverse trend could be caused by the presence of *Lactobacillus* spp. in the women with intermediate vaginal flora and Nugent scores of 5 and 6. Unfortunately, we were not able to investigate this further, as detailed information on the vaginal microbiota was not available in these women. Moreover, the association of *Lactobacillus iners* and *Lactobacillus gasseri* with intermediate vaginal flora and BV, as well as the negative influence of *Lactobacillus iners* on PTD in pregnant women should be considered [22,23].

On comparing the Lactobacilli and Non-Lactobacilli groups, we observed significant differences for PTD and mean birth weight, although the groups had the same Nugent score of 4. The greatest differences were found for women who delivered between 23 + 0 and 31 + 6 gestational weeks and for those whose infants had a birth weight of 500 to 1499 g. This was confirmed in a multiple regression model after adjusting for parity as the only significant confounder (Table 4). The probabilities of delivery earlier than 37 + 0 gestational weeks and a birth weight of less than 2500 g were higher in the Non-Lactobacilli group than in the Lactobacilli group. Hence, women with intermediate vaginal flora and a Nugent score of 4 had high risks of PTD and delivery of infants with low birth weight when vaginal *Lactobacillus* spp. were absent. From a clinical perspective, this finding may encourage treatment with *Lactobacillus* spp. in asymptomatic women with a Nugent score of 4 and the total absence of any bacterial colonisation. Moreover, the finding that women with intermediate vaginal flora, apart from those with BV, are at risk for PTD supports the implementation of routine antenatal screening programmes to detect abnormal flora and initiate treatment, which will help to improve obstetrical outcomes [13,24].

We attempted to detect a shift towards BV later in pregnancy by evaluating vaginal follow-up smears, as this shift could be responsible for the high risk of PTD in the Non-Lactobacilli group. However, after evaluating the available follow-up smears, we found that the rates of BV development were similar in the Lactobacilli and Non-Lactobacilli groups (9% vs. 7.8%, Fig 2). We assumed that the high rate of normal flora in the Non-Lactobacilli group might have been achieved through improved colonisation with vaginal *Lactobacillus* spp. in the total absence of any bacteria. However, the colonisation might be impeded when non-Lactobacilli rods are present, and this would explain the low rates of normal flora in follow-up smears of the Lactobacilli group. In 2014, Honda et al. [20] observed a shift of the vaginal flora from normal to intermediate on routine infection screening in the second trimester among women who later experienced PTD. The authors suggested that PTD was associated with intermediate flora, rather than with BV, and that this shift might be the reason for the failure in the prevention of PTD reported in several studies [20]. We do not believe that a shift towards BV can explain our findings, although the limited availability of follow-up smears in our study, and the overall small number of women who underwent consequent follow-up should be taken into account.

To our knowledge, this is the first paper evaluating the role of *Lactobacillus* spp. in the intermediate vaginal flora, apart from BV [11,12,15]. Although our study is one of the largest studies to evaluate the role of *Lactobacillus* spp., our findings should be interpreted with caution considering the retrospective design of the study. It is possible that factors other than infection that occurred later in pregnancy contributed to the PTD rate in the Non-Lactobacilli group. Although vaginal infection is a major causative factor of PTD, underlying factors, such as diabetes, hypertension, and obesity, should be considered [18]. In view of the high PTD rate (32.7%) among women with a Nugent score of 4, it should also be considered that our study was performed in a high-risk obstetrical setting. We previously published that the overall PTD rate within our asymptomatic screened population, including women with intermediate vaginal flora, was 9.7% [13]. Analysis of the role of *Lactobacillus* spp. in women with Nugent scores of 5 and 6, as well as analysis according to the various subtypes of vaginal Lactobacilli, should be performed in future studies. Moreover, more comprehensive scoring systems, such as that of Ison and Hay [25], should also be considered when interpreting our results.

## Conclusion

The present study demonstrated an association between intermediate vaginal flora and the multifactorial mechanism of PTD. In particular, women with a Nugent score of 4 and the absence of any bacterial colonisation, including that by *Lactobacillus* spp., showed an increased risk of PTD and low birth weight upon multiple regression analysis. These findings indicate that vaginal *Lactobacillus* spp. play an important role in the intermediate vaginal flora and that their absence may be associated with a pathological condition. In particular, among women with intermediate vaginal flora and a Nugent score of 4, those without *Lactobacillus* spp. should be distinguished from those with *Lactobacillus* spp. Further prospective studies are required to determine whether a reassessment of the Nugent scoring system in this regard is needed.

## Supporting Information

S1 Dataset (XLS)

## Abbreviations

BV: bacterial vaginosis
CI: confidence interval
*Lactobacillus* spp.: *Lactobacillus* species
MD: mean difference
OR: odds ratio
PTD: preterm delivery
SD: standard deviation
